# Psychometric properties of the Children’s Revised Impact of Events Scale (CRIES) with Bangladeshi children and adolescents

**DOI:** 10.7717/peerj.536

**Published:** 2014-08-26

**Authors:** Farah Deeba, Ronald M. Rapee, Tania Prvan

**Affiliations:** 1 Centre for Emotional Health, Department of Psychology, Faculty of Human Sciences, Macquarie University, NSW, Australia; 2 Department of Statistics, Faculty of Science, Macquarie University, NSW, Australia

**Keywords:** Assessment, Trauma, Post-traumatic stress, Children, Bangla, Bangladesh, Adolescents, PTSD

## Abstract

Identification of possible cases suffering post-traumatic stress disorder (PTSD) is important, especially in developing countries where traumatic events are typically prevalent. The Children’s Revised Impact of Events Scale is a reliable and valid measure that has two brief versions (13 items and 8 items) to assess reactions to traumatic events among young people. The current study evaluated the psychometric properties of both versions of the CRIES in a sample of 1,342 children and adolescents aged 9–17 years (*M* = 12.3 years, *SD* = 2.12) recruited from six districts of Bangladesh. A sub-group of 120 children from four schools was re-tested on the measures within 3.5 weeks. Confirmatory factor analysis supported factor structures similar to those found in other studies for both versions of the CRIES. Multiple group confirmatory factor analysis showed gender and age-group differences within the sample, supporting established age and gender differences in prevalence of PTSD symptoms. Analyses also indicated moderate to excellent internal consistency and test-retest reliability and clear discriminant and convergent validity. These data support use of both the CRIES-13 and CRIES-8 to provide quick and psychometrically sound assessment of symptoms of PTSD among children and adolescents from Bangla-speaking communities.

## Introduction

In the aftermath of exposure to traumatic events, about 70% of children develop symptoms of Post-Traumatic Stress Disorder (PTSD) within the first month after the incident (Aaron, Zaglul & Emery, 1999) and almost 20–30% will meet full diagnostic criteria for PTSD within the first 12 months (Dyregrov & Yule, 2006; Schnurr et al., 2007). When children with PTSD are left untreated, the disorder can persist for years limiting their psychosocial functionality and increasing risk for other disorders (Bolton et al., 2000; Weber et al., 2008; Yule et al., 2000). Trauma can also produce marked neurobiological consequences and impaired cognitive development that can reduce academic and social performance in a young person’s life (Teicher et al., 2003; Yasik et al., 2007). In the long run, the impact on individual levels of productivity across the life-span increases burden on the whole society. To help reduce this long-term impact, early identification of post-traumatic stress reactions is very important (Cohen et al., 2010).

Unfortunately traumatic events are more common in the lives of children from developing or low and middle income countries than those of developed countries creating a greater vulnerability to mental health problems (Matzopoulos et al., 2008; Patel & Kleinman, 2003; Whetten et al., 2011). Despite the frequency of traumatic events in developing countries, a lack of standard assessment and screening tools to identify young people suffering distress is a common problem that limits the efficiency of service delivery. Direct interviews and more importantly, structured diagnostic interviews require resources that are simply not available in most developing countries, especially following large-scale traumas (e.g., Ahmed et al., 2011; Rousham, 1996). Therefore, increased availability of free and well validated measures that have been translated and evaluated in developing countries, is vitally important.

Bangladesh is one developing country where children’s lives are continually affected by a variety of traumatic events. The range of traumatic events includes natural traumas, accidents, and man-made traumas. Bangladesh is well known to the rest of the world for its frequent natural disasters and has been identified as the country with the highest number of natural disasters in the world (Government of the People’s Republic of Bangladesh, 2008). Young people are typically most severely affected by natural disasters through death, disability, loss of family, and displacement. A large number of subsequent problems add to the vulnerability of children including, neglect, abuse, human trafficking, or loss of education (UNICEF, 2008). In addition to frequent natural traumas, large numbers of children in Bangladesh are traumatised each year due to a variety of accidents (Linnan et al., 2007). More than 82 children die every day in Bangladesh as a result of unintentional traumatic injury, one of the highest rates in the world (Rahman, 2005). Many young people also face a range of man-made traumatic events, including trafficking (Ali, 2005), rape (Al-Azad et al., 2012), acid attack (Zafreen et al., 2010) and many other serious forms of violence (UNICEF, 2012).

Despite mounting recognition of the quantity of traumatic events in the lives of young Bangladeshi people which point to the need for both physical and mental health support, there are few reliable data in the country regarding childhood post-traumatic stress reactions. In one large-scale survey, children showed higher levels of aggression and enuresis following a major flood compared to levels before the flood (Durkin et al., 1993). Similarly, high levels of traumatic reactions were reported following a tornado (13 May 1996) where among 150 victims (both adults and children), 66% were found to be psychologically traumatized (Choudhury, Quraishi & Haque, 2006).

Given the high frequency of trauma in the country and the particular vulnerability of children, it is highly likely that a significant proportion of Bangladeshi children will suffer post-traumatic stress reactions. Yet no formal reports are currently available that quantify levels of traumas in the country. This gap in knowledge partly reflects the decreased importance given by policy makers and the public to mental health issues, combined with a lack of resources to address these problems. Being able to quantify psychological reactions to trauma through the use of brief, valid and easily administered self-report measures would assist in redressing this situation (Ohan, Myers & Collett, 2002). Availability of such measures will not only be useful for epidemiological surveys, but would also be of value for clinical practice or research.

Well-developed self-report screening tools to assess children’s psychological symptoms require several key characteristics. Such tools need to be brief to ensure that they can be quickly completed with minimum disruption to the individual (Brewin et al., 2002; Stallard, Velleman & Baldwin, 1999) and items need to be easily understood by children (Yule, 1992). Within communities with few resources, it is also important that instruments are easily administered and able to be scored by non-professionals (Brewin et al., 2002). Several widely used measures of post-trauma reactions among children fail to meet all of these criteria. Among the measures of childhood PTSD, the Children’s Revised Impact of Events Scale (CRIES; Children and War Foundation, 2005) fulfils the criteria for good screening instruments and has been used across a large number of countries and cultures (both Western and Eastern). This measure has been translated into more than 15 languages and has been used in a number of countries following various large and small scale disasters. Examples include its use with children and adolescents affected by war in Bosnia-Hercegovina (Smith et al., 2001), earthquakes in Greece (Giannopoulou et al., 2006b) and China (Zhao et al., 2009), tsunami in Sri-Lanka (Ketumarn et al., 2009), and also following road-traffic accidents or other emergency medical injuries in the UK (Perrin, Meiser-Stedman & Smith, 2005) and Australia (Kenardy, Spence & Macleod, 2006). The CRIES has shown good reliability, satisfactory face and construct validity, a stable factor structure, and has been used to screen large samples of at-risk children following a wide range of traumatic events (Smith et al., 2003). Particular advantages of the CRIES include its brevity, simple scoring that requires minimal training, clear adherence to PTSD diagnostic criteria in the DSM, and it can be used even with children as young as five (e.g., Malmquist, 1986). Above all, the CRIES is a free resource that is made available through the website of the Children and War Foundation, a Norwegian-based non-profit organisation.

Although the original 15-item CRIES (Malmquist, 1986; Yule & Williams, 1990) was designed to cover the three components of PTSD, intrusion, avoidance, and emotional numbing, confirmatory factor analyses failed to support a three-factor structure. Several studies found that most items loaded onto two factors (intrusion and avoidance), and several items did not load on either factor or on more than three factors (Dyregrov, Kuterovac & Barath, 1996; Sack et al., 1998; Yule, Bruggencate & Joseph, 1994). In response, Yule (1997) removed seven items from the original scale and developed a short, eight-item version, the CRIES-8 comprised of the two factors, intrusion and avoidance. Finally, to better reflect DSM-defined PTSD symptoms (American Psychological Association, 2000), five additional items were added to the CRIES-8 to represent the third cluster of PTSD symptoms, arousal (Perrin, Meiser-Stedman & Smith, 2005; Smith et al., 2003). These additional items completed the CRIES-13 and the three sub-scales were labelled Intrusion, Avoidance and Arousal (Children and War Foundation, 2005).

The factor structure of the CRIES-13 across several studies has been slightly inconsistent, variously showing a two-factor structure (intrusion and arousal vs avoidance) (Chen et al., 2012), three distinct but inter-correlated factors (intrusion, arousal and avoidance) (Zhang et al., 2011), and a three-factor structure loading onto a single higher order factor (intrusion, arousal, and avoidance loaded onto PTSD) (Giannopoulou et al., 2006b). Nonetheless, psychometric properties (for instance, reliability and validity, please see method for detail) for both the CRIES-8 and CRIES-13 have been solid.

Both versions of the CRIES have shown good utility when used as screening tools for children exposed to traumatic events (Dow et al., 2012; Perrin, Meiser-Stedman & Smith, 2005). A cut-off score of 17 on the CRIES-8 and a cut-off score of 30 on the CRIES-13 were found to produce the best balance between sensitivity (.94 and .91) and specificity (.59 and .65) to identify PTSD in a group of children referred for assessment, and sensitivity (1.0 and .86) and specificity (.71 and .73) to identify PTSD in a group of children assessed in a hospital accident and emergency department (Perrin, Meiser-Stedman & Smith, 2005).

Although symptoms of PTSD and post-traumatic reactions have been argued to be universally consistent (Giannopoulou et al., 2006b), it remains possible that different language and cultural groups will demonstrate differences in perceptions and reactions to a given event (e.g., Anthony & Michael, 2004). Given the importance of having a brief and inexpensive instrument to assess post-traumatic reactions among young people in Bangladesh, the present study aimed to establish the psychometric properties (that is, confirmatory factor analyses, internal consistency, reliability and validity) of the CRIES-8 and CRIES-13 in a large sample of children and adolescents from Bangladesh.

## Methods

### Participants

A total of 1,342 children and adolescents from a larger sample of 1,383 participants for a different study (F Deeba & RM Rapee, 2014, unpublished data) who reported on at least 90% of the items of the CRIES 13 were included in the current sample (Males = 467, 34.68% and Females = 875, 65.32%). Children were recruited from 10 schools (primary, secondary and high) and 39 social support centres for children with traumatic experiences, across rural and urban (slum and non-slum) areas from the six districts of Bangladesh. The social support services participating in the study comprised a broad group of organizations, both government and non-government that aimed to provide social welfare (for example, shelter, educational, health, legal and other support) for disadvantaged or vulnerable children in residential or non-residential forms. We provided detailed information about inclusion and exclusion criteria to social support staff and class teachers, before conducting any assessment session. Support staff and teachers then selected children for the assessment session based on this information if they believed that the child did not suffer psychosis or attention deficit hyperactivity disorders, and had no major vision, hearing or intellectual problems. Children from schools comprised a group of community children (*N* = 562, 41.88%) while those who were collected through support centres run by government and non-government organizations constituted an “at-risk” group (*N* = 780, 58.12%).

A wide variety of traumatic events were reported by children, including natural disasters (e.g., flood, cyclone, tornado, avalanches, arsenic exposure, suffering from terminal disease, and others), accidents (e.g., hit by a road transport vehicle, boat or launch accidents, train/plane accidents, building collapse, fire, fall from highs, drowning, explosions and others) and man-made traumas (e.g., hit by others, suffocated, attempt to kill, acid attack, bombing, verbal abuse, bullying (peers), threat to hurt, stalking, sexual abuse (penetrative and non-penetrative), trafficking, mugged/robbed, and others). The majority of children in both groups had experienced at least one trauma (see Table 1). The two sub-groups of the sample differed significantly on the number of traumatic events experienced, *χ*^2^(4, *N* = 1,342) = 27.37, *p* < .001. Over half of the children in at-risk group had 7 and more traumatic experience, whereas the community children were just under 40% of 7 and more traumatic events exposure (for more detail see, F Deeba & RM Rapee, 2014, unpublished data).

**Table 1:** Demographic variables within the two sub-samples.

	Community	At-risk
	(*N* = 562)	(*N* = 780)
Mean Age (SD)	12.27 (1.89)	12.26 (2.26)
Males (n, %)	228 (40.56)	239 (30.64)
**Educational and Work status (*n*, %)**		
Education	547 (97.32)	450 (57.69)
Work	1 (0.18)	58 (7.44)
Education & work	14 (2.50)	240 (30.77)
Others	–	32 (4.10)
**Religion (*n*, %)**		
Muslim	474 (84.34)	735 (94.23)
Hindu	86 (15.30)	36 (4.62)
Others	2 (0.36)	9 (1.05)
**Frequency of traumatic events** **experience (% within group)**		
Single event	28 (4.98)	46 (5.90)
2–3 events	109 (19.40)	133 (17.05)
4–6 events	213 (37.90)	206 (26.41)
7 to more events	212 (37.72)	395 (50.64)

Children from the social support centres mostly lived in slum areas or shelter homes. Participation from children approached in social support centres (90%) was higher than among children from the community group (75%). The age range of the sample was 9–17 years (mean age = 12.3 years, *SD* = 2.12). There were 756 (56.34%) children aged 9–12 years and 586 (43.66%) adolescents aged 13–17 years. Demographic information about the two sub-samples is given in Table 1.

A subsample of 135 children (Males = 49, 40.83%) from four schools in Dhaka completed the same measures 3–4 weeks (average 3.5 weeks) following initial assessment. Their mean age was 12.92 years (*SD* = 1.96). Among them 120 children completed 90% of the total items and were included in the analysis.

### Measures

#### Children’s Revised Impact of Events Scale-13 (CRIES-13)

As described above, the CRIES-13 and CRIES-8 (Children and War Foundation, 2005) share the same eight items that constitute two subscales, Intrusion and Avoidance, and the CRIES-13 includes an additional five items that constitutes a third sub-scale, Arousal. Items are scored on a non-linear scale as follows: 0 (not at all), 1 (rarely), 3 (sometimes) and 5 (often). Scores range from 0 to 40 for the CRIES-8 and 0 to 65 for the CRIES-13, and higher scores indicate more PTSD symptoms.

Internal consistencies range from .75 to .87 for the total CRIES-13, .75–.84 for the total CRIES-8 and for the three subscales; Intrusion: .70–.90; Avoidance: .62–.82 and Arousal .60–.74 (Dyregrov, Kuterovac & Barath, 1996; Giannopoulou et al., 2006a; Lau et al., 2013; Smith et al., 2003; van der Kooij et al., 2013; Yule, Bruggencate & Joseph, 1994; Zhang et al., 2011). Test retest reliability up to 7-day is good for the total CRIES-13 (*r*′*s* = .76–.85) (Panter-Brick et al., 2011; Verlinden et al., 2014), and *r* = .75 for CRIES-8 (Verlinden et al., 2014). However, it is less acceptable for the subscales; Intrusion *r* = .58; Avoidance: *r* = .68 and Arousal: *r* = .53 (van der Kooij et al., 2013).

Validity for both the CRIES-8 and CRIES-13 has also proven satisfactory (Perrin, Meiser-Stedman & Smith, 2005). For instance, children experiencing symptoms of PTSD have been shown to score higher on the CRIES-8 than children without PTSD (Stallard, Velleman & Baldwin, 1999). Similarly, in a large sample of children affected by war (*N* = 2,976) in Bosnia-Hercegovina, scores on the CRIES-13 and all subscales showed small positive correlations (*r* = .05–.36) with self-reported level of traumatic event exposure, and depression (Smith et al., 2002) and also with ratings of children’s distress from parents and teachers and with mothers’ levels of trauma exposure and distress (Smith et al., 2001).

#### Spence Children’s Anxiety Scale-20 (SCAS-20)

SCAS-20 (SH Spence, pers. comm., 2010) is a simple, brief self-report questionnaire to assess symptoms of anxiety. The SCAS-20 is a short form of the more commonly used 38-item SCAS (Spence, 1998). Items are rated on a 4-point Likert-type scale as 0 (never), 1 (sometimes), 2 (often) and 3 (always) and summed to obtain a total score where higher scores indicate higher levels of anxiety. Items for the short version were selected from factor analyses of the full version (Spence, 1998; Spence, Barrett & Turner, 2003). Although the psychometric properties of the short version have not yet been published, an unpublished evaluation of the SCAS-20 demonstrated strong internal consistency of .89 (Coysh, 2011). The psychometric properties of the SCAS-20 among a group of Bangladeshi children and adolescents showed good internal consistency (Cronbach’s alpha .84) and satisfactory construct validity for the scale (F Deeba, RM Rapee & T Prvan, 2014, unpublished data).

#### Short Moods and Feelings Questionnaire (SMFQ)

SMFQ (Angold et al., 1995) was developed to identify DSM-IV-based signs and symptoms of depressive disorders in children and adolescents aged 6–17 years. The scale is scored on a 3-point Likert-type response scale 0 (Never); 1 (Sometimes true) and 2 (Always true). The total score is the sum of all items providing possible scores ranging from 0 to 26 with higher scores reflecting lower mood and risk of clinical level depression. The SMFQ has been shown to comprise a single factor and has good criterion-related validity and discriminant validity to identify clinical levels of depression in children and adolescents (Angold et al., 1995; Thapar & McGuffin, 1998). Cronbach’s alpha for the SMFQ has been reported ranging from .87 to .90 (Angold et al., 1995). For the Bangladeshi children and adolescents, Cronbach’s alpha was strong at .80 (F Deeba, RM Rapee & T Prvan, 2014, unpublished data).

### Translation of measures

Standard guidelines accepted for the successful translation of instruments for research purposes (e.g., Brislin, 1986) were used. The bilingual investigator translated the English version of the CRIES to Bangla. Then another bilingual professional psychologist not associated with the measure translated it back from Bangla to English. Back translation was checked by the second author of the study, who is a native English speaker. Differences in the two versions were resolved by joint agreement of both translators.

### Procedure

**Figure 1:**
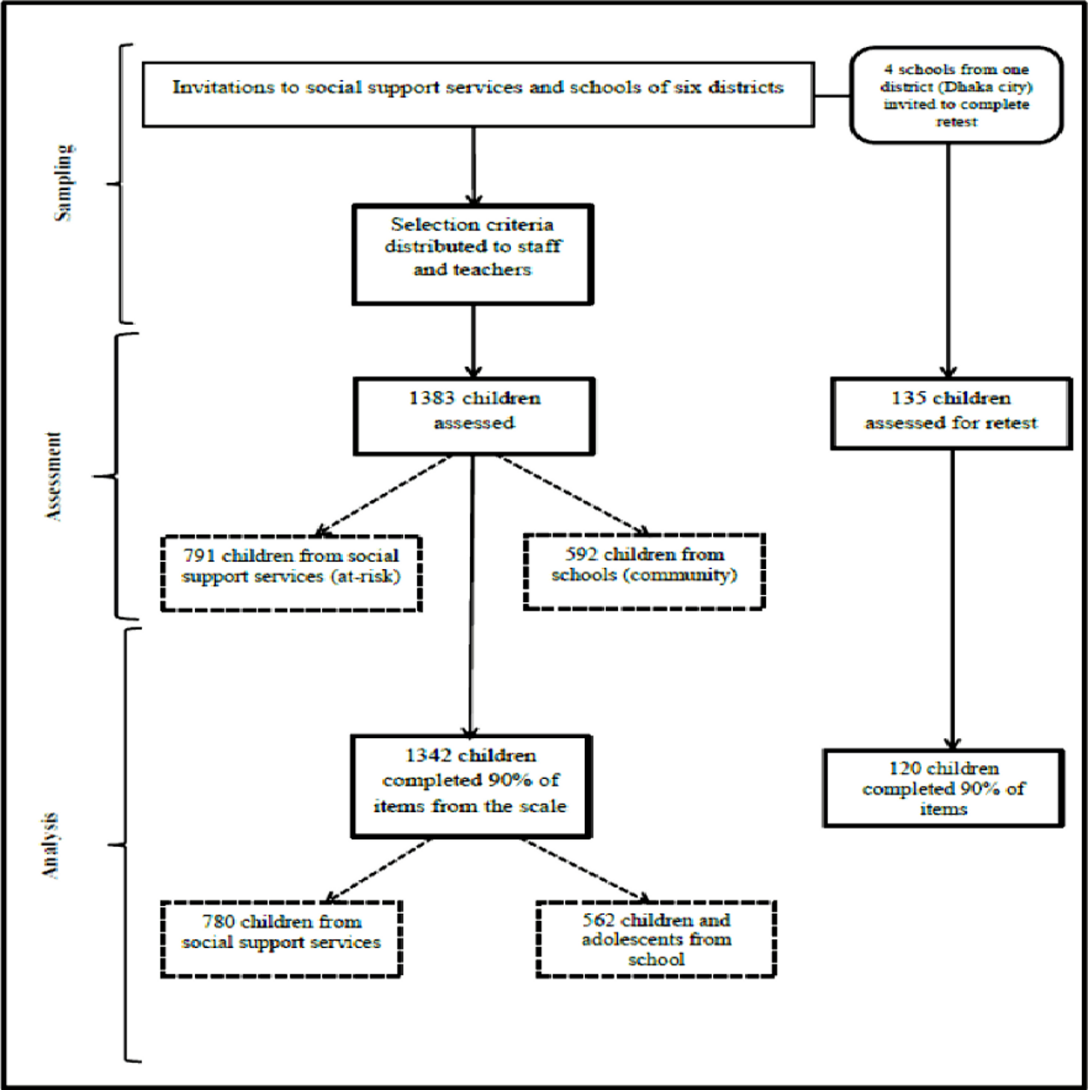
Flow-chart to demonstrate sample sizes of participants in the study at different steps.

Ethical issues in the study were reviewed and approval granted by the Macquarie University Human Research Ethics Committee (Ref no. 5201001017 dated 5/11/2010). Written permission was sought from every institution and organization where the study was to be conducted. Individual consent was collected for each child from their parents or caregivers and children provided assent, before all assessment tasks. Issues of voluntary participation, freedom to respond independently, confidentiality and seeking clarification during assessment were discussed with the children at the beginning of the assessment sessions. Assessments were conducted at a time decided by the organisation, in groups of up to 30 children unless children were aged less than 12 years or were illiterate. In such cases the maximum number of children in the assessment group was 10 and items were read aloud by the researcher (along with items for another study, see F Deeba & RM Rapee, 2014, unpublished data). A psychology post-graduate research student was recruited to assist the first author to conduct assessment sessions. The assistant was trained in administering the measures and the ethical issues involved with assessment. The test-retest reliability of the measure was checked after 3.5 weeks following the same procedure stated above with 120 school children from four schools in the capital city. For clarity, distributions of participants and samples sizes for particular analyses are shown in Fig. 1.

### Statistical analysis

All analyses were conducted using SPSS V.21 and its extension AMOS V.21. Missing data were handled by the Person Mean Substitution method (PMS, Downey & King, 1998) due to the non-linear scoring of the items. Confirmatory Factor Analysis (CFA) with the 13-item CRIES compared three different measurement models based on previous studies (e.g., Giannopoulou et al., 2006b; Smith et al., 2003; Zhang et al., 2011). The models were: Model 1—single-factor (PTSD) model, Model 2—two inter-correlated latent factors, [(i) intrusion/arousal and (ii) avoidance], Model 3—three inter-correlated latent factors [(i) intrusion (ii) avoidance and (iii) arousal] and Model 4—three latent factors [(i) intrusion (ii) avoidance and (iii) arousal] loading onto a single higher-order factor (PTSD). We did not run a separate CFA for the CRIES-8 since the items and subscales are embedded in the CRIES-13.

Maximum Likelihood (ML; Byrne, 2010) tests were used on the whole sample (*N* = 1,342) for model identification, and then two separate multiple group confirmatory factor analyses (MCFA) were run on the best fitting model to evaluate model invariance between gender and age-groups (younger/older) by group affiliation (community and at-risk) following Byrne (2004). Standardized parameter estimates are reported. Model fit statistics in the present study were selected from suggestions by Jackson, Gillaspy & Purc-Stephenson (2009) and cut-offs for model fit indices were selected as per Kline (2005) and Worthington & Whittaker (2006) as best for clinical measures. These included the goodness-of-fit index (GFI), for which values greater than .90 are acceptable (Hu & Bentler, 1999), the comparative fit index (CFI), and the Tucker-Lewis index (TLI) where values equal to or greater than .90 are considered a good fit (Dumenci & Achenbach, 2008). To observe differences between observed and predicted covariances, the Root Mean Square Error of Approximation (RMSEA) was chosen. RMSEA values less than .06 (Hu & Bentler, 1999) or .08 (Dumenci & Achenbach, 2008) have been proposed as indicating a good–fitting model, though RMSEA values of .06–.08 are often reported as acceptable or reasonable rather than good (Kline, 2005; McDonald, 2002). To determine the optimal and most parsimonious model, the Akaike Information Criterion (AIC; Akaike, 1973) and Bayes Information Criterion (BIC; Schwarz, 1978) were checked as per suggestions by Bozdogan (1987) that lower values indicate better fit. Factor loadings on items found not to be invariant across groups in MCFA were reported.

Reliability of the measures was evaluated by examining both internal consistency and test-retest reliability. Convergent validity was determined by calculating Pearson’s product moment correlation coefficients between the CRIES, SCAS-20 and SMFQ and discriminant validity was determined by comparing scores from at-risk children (from support services) and community children (from schools). Finally, to understand the influence of age and sex on the measure, 2 (gender) X 2 (age group) ANCOVAs were conducted on the CRIES-13 and CRIES-8 total and sub-scale scores controlling for group affiliation (at-risk and community children).

## Results

### Confirmatory factor analysis

All hypothesised models for the CRIES were identified in the measurement model specification analyses. Results are reported in Table 2. The *χ*^2^ value was significant at *p* < .001 for all the models which is common for any large sample (Byrne, 2010), therefore, we considered the other fit indices to decide the best structural model for both the long and short versions of the measure.

**Table 2:** Fit indices for the four hypothesised models on the CRIES-13 based on the total sample.

	*χ* ^2^	*df*	*p*	GFI	CFI	TLI	RMSEA (95% CI)	AIC	BIC
Model 1	363.04	65	.001	1.00	.84	.81	.06 [.05–.06]	415.04	550.29
Model 2	206.11	64	.001	.98	.91	.92	.04 [.04–.05]	260.10	400.55
Model 3	166.33	62	.001	.98	.94	.93	.04 [.03–.04]	224.33	375.18
Model 4	166.33	62	.001	.98	.94	.93	.04 [.03–.04]	224.33	375.18

**Notes.**

CRIES-13 Children’s Revised Impact of Events Scale, 13-item version

**Figure 2:**
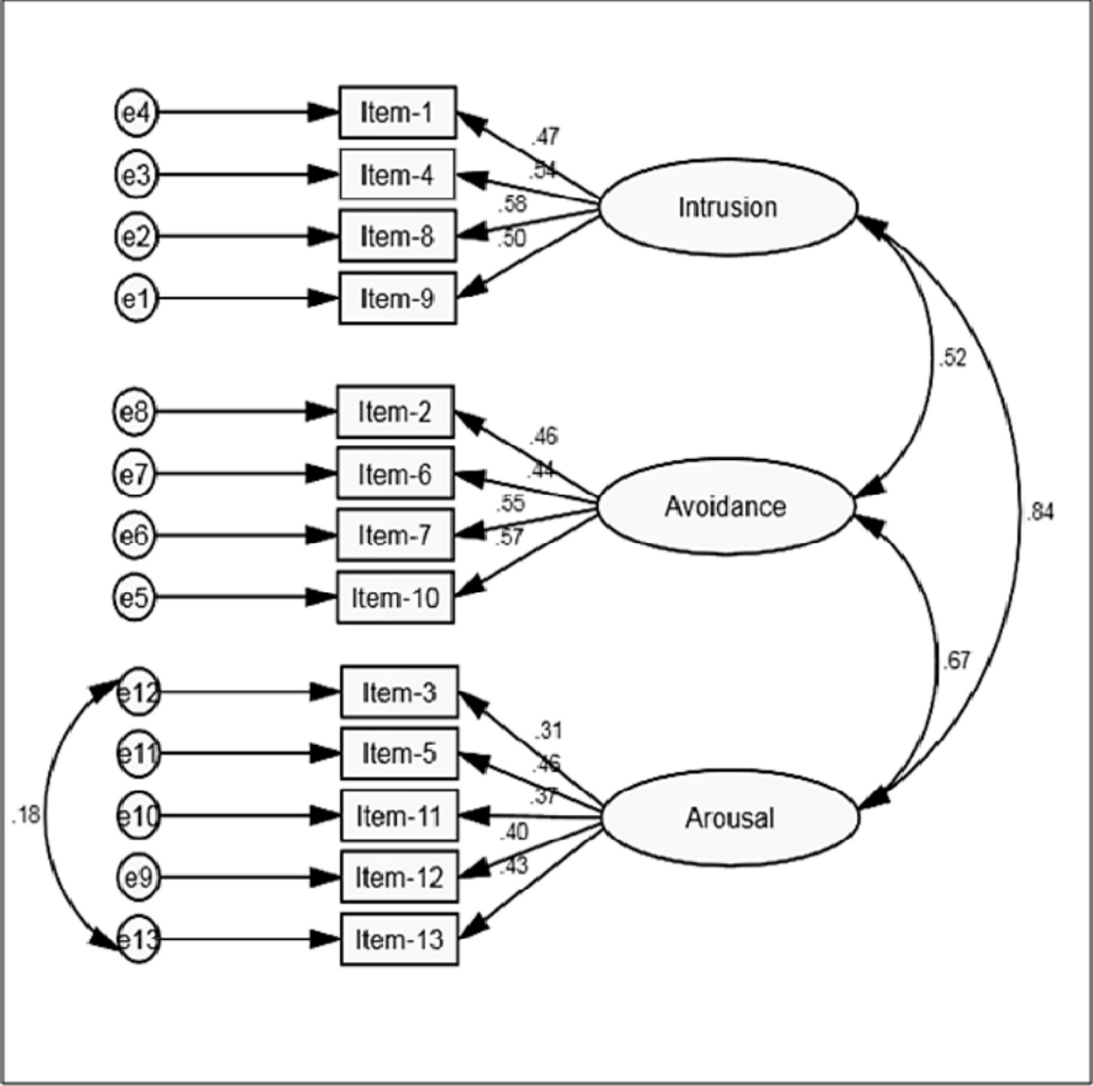
Three-factor solution for the CRIES-13 with total group (*N* = 1,342).

As can be seen in Table 2, the modification indices for Models 3 and 4 were identical and these two models for the CRIES-13 produced a better fit than either Model 1 or Model 2. Therefore, based on the “Principle of Parsimony” (Bollen, 1989), we selected Model 3 (see Fig. 2), with three correlated factors as the most suitable representation of the factor structure of the CRIES-13. The correlations shown by the double headed arrows between the three factors also represent the correlations between the three sub-scales of the measure. All items were positively correlated and correlation coefficients for the three latent factors were moderate to strong (.52–.81). All items had standardized estimates that ranged from .36–.58. None of the multiple *R*^2^ values were below .02 although Item 3 (*Do you have sleep problems*?), Item 11 (*Do you get easily irritable?*) and Item 12 (*Are you alert and watchful even when there is no obvious need to be?*) did not load strongly on their relevant latent factor (arousal; *R*^2^ = .13–.16). Factor loadings for items on intrusion (.47–.58) and avoidance (.44–.57) were generally higher than for arousal (.36–.47). Based on the covariance matrices, a free parameter was needed between the error terms of Item 3 (*Do you have difficulties paying attention or concentrating?*) and Item 13 (*Do you have sleep problems?*). When these error terms were permitted to vary together (constrained under the same latent variable) improvements were shown in the fit for Model 3: CMIN = 132.33, DF = 61, GFI =.98, CFI =.96, TLI =.95, RMSEA =.03 (95% CI [.02–.04]), AIC = 192.22, BIC = 348.28. Therefore, it was evident that a slightly modified Model 3 provided the best factor structure for the measure.

Consequently we decided to use the modified Model 3 as the hypothesised baseline model to examine model invariance with gender and age-group, within each sample (community/at-risk). Initially, we tested model invariance with the four different groups of gender (community boy, community girl, at-risk boy and at-risk girl) and then with the age-groups (community-younger, community older, at-risk younger, and at-risk older). The results of the model invariance tests for the baseline model and constrained models are reported in Table 3 with both gender and age-groups. Results failed to demonstrate complete structural invariance across gender and age, which is not unusual. Importantly, however, for all models (i.e., unconstrained, constrained with measurement weights, structural covariances and measurement residuals) tests for the modified Model 3 yielded an acceptable range of model fit indices for each subgroup. Factor loadings for individual items on the three factors (Intrusion, Avoidance and Arousal) were reasonable for community males (.27–.64), community females (.24–.64), at-risk males (.22–.59), and at-risk females (.26–.64) and also for community younger (.29–.55), community older (.11–.67), at-risk younger (.15–.60), and at-risk older (.35–.65) children. Hence these results indicate that the modification of Model 3 provided the best fit for the data consistently across all subgroups.

**Table 3:** Multiple group analyses for model invariance for Model 3 of CRIES-13 with four groups of community and at-risk children by gender and age-groups.

	*χ* ^2^	*df*	*p*	RMSEA (95% CI)	Δ*χ*^2^	Δ*df*	Statistical significance
**Four groups by gender** ^a^							
Model A: Unconstrained	366.13	244	.001	.019 [.015–.023]	–	–	–
Model B: Measurement weights	140.93	274	.001	.019 [.015–.023]	44.80	30	.040
Model C: Structural covariances	432.94	292	.001	.019 [.015–.023]	66.81	48	.038
Model D: Measurement residuals	524.56	334	.001	.021 [.017–.024]	158.43	90	.001
**Four groups by age-group** ^b^							
Model A: Unconstrained	348.51	244	.001	.018 [.013–.022]	–	–	–
Model B: Measurement weights	394.54	274	.001	.018 [.014–.022]	46.03	30	.01
Model C: Structural covariances	437.32	292	.001	.019 [.015–.023]	88.82	48	.01
Model D: Measurement residuals	564.31	334	.001	.023 [.019–.026]	215.80	90	.001

**Notes.**

a Community-boy, community-girl, at-risk-boy and at-risk girl.

b Community-younger, community-older, at-risk-younger and at-risk older.

CRIES Children Impact of Event Scale

**Table 4:** Internal consistency (Cronbach’s alpha) of two versions of CRIES and three sub-scales of the scale with different sub-groups of the sample.

Sub-groups of sample		CRIES-13	CRIES-8	Intrusion	Avoidance	Arousal
**By types of organizations**	Community	.70	.62	.53	.60	.47
	At-risk	.72	.67	.63	.55	.49
**By gender**	Males	.68	.60	.57	.53	.45
	Females	.74	.68	.62	.59	.51
**By age-groups**	Younger	.70	.61	.56	.54	.50
	Older	.75	.69	.65	.62	.50

**Notes.**

CRIES-13 Children Impact of Event Scale-13 CRIES-8 Children Impact of Event Scale-8

### Reliability

Cronbach’s alpha for the total CRIES-13 was alpha =.74 and for the total 8-item version was alpha =.70. Internal consistencies for the three subscales of the two versions of the CRIES were moderate: Intrusion (alpha =.60), Avoidance (alpha =.58) and Arousal (alpha =.50). Cronbach’s alphas within the different sub-groups are reported in Table 4.

Pearson product moment correlation coefficients were calculated between questionnaire scores on the two versions of the measure separated by 3.5 weeks within a sub-group of community children (*N* = 120). Results showed a significant moderate relationship for the total score on the CRIES-13 (*r* = .72, *p* < .001), and for the CRIES-8 (*r* = .62, *p* < .01). Test-retest reliability for each sub-scale was also moderate (Intrusion .67 [*p* < .01], Avoidance .50 [*p* < .01], and Arousal .67 [*p* < .01]).

### Validity

#### Convergent validity

The relationship between scores on the two versions of the CRIES and the SCAS-20 and SMFQ were calculated. All correlations were positive and significant at *p* < .01. Specifically the following correlations were demonstrated with the SCAS-20: CRIES-13 (*r* = .58), CRIES-8 (*r* = .48), Intrusion (*r* = .36), Avoidance, (*r* = .20), Arousal (*r* = .41). Similarly, correlations with the SMFQ were as follows: CRIES-13 (*r* = .42), CRIES-8 (*r* = .34), Intrusion (*r* = .44), Avoidance, (*r* = .34), Arousal (*r* = .53).

#### Discriminant validity

Scores on the CRIES-13 and CRIES-8 (as well as each subscale) were compared between the two samples of children: community children (selected primarily from schools in the general community) and at-risk children (selected from social support centres). In each case, at-risk children scored significantly higher on the various measures than community children (all *p*’s <.01), see Table 5.

**Table 5:** Means, SDs of CRIES-13, CRIES-8 and the three sub-scales, first on the total sample and then comparing the two sub-samples.

Measure	Total (*N* = 1,342)	Community (*N* = 562)	At-risk (*N* = 780)	*t*-tests comparing community and at-risk samples
	M (*SD*)	M (*SD*)	M (*SD*)	
CRIES-13	25.12 (11.87)	22.08 (10.97)	27.30 (12.02)	*t*(1340) = −8.15, *p* < .001
CRIES-8	17.11 (8.35)	15.27 (7.88)	18.43 (8.44)	*t*(1340) = −6.96, *p* < .001
Intrusion	8.59 (4.86)	7.61 (4.49)	9.30 (4.99)	*t*(1340) = −6.39, *p* < .001
Avoidance	8.51 (5.44)	7.66 (5.37)	9.13 (5.41)	*t*(1340) = −4.91, *p* < .001
Arousal	8.00 (5.28)	6.80 (4.78)	8.87 (5.45)	*t*(1340) = −7.19, *p* < .001

**Notes.**

CRIES-13 13-item Children’s Revised Impact of Events Scale CRIES-8 8-item Children’s Revised Impact of Events Scale

### Demographic differences on CRIES

Total scores on the CRIES-13 and CRIES-8 and also each sub-scale were compared between gender and age groups using a series of 2×2 ANCOVAs,^1^1Similar analyses were conducted to examine subgroup differences separately for the two samples, community and at-risk children. Results were very similar to those for the total sample and therefore only the total sample analyses are reported here. with the two samples (community and at-risk) included as a covariate. On the CRIES-13, there were significant main effects for gender, *F*(4, 1,337) = 17.99, *p* < .001, ${\eta }_{p}^{2}=.01$ and age-group, *F*(4, 1,337) = 26.65, *p* < .001, ${\eta }_{p}^{2}=.02$, but the interaction between gender and age group was not significant, *F*(4, 1,337) = .001, *p* = .94, ${\eta }_{p}^{2}=.01$. Similarly, for the CRIES-8, there were significant main effects for gender, *F*(4, 1,337) = 9.37, *p* < .01, ${\eta }_{p}^{2}=.01$, and age-group, *F*(4, 1,337) = 25.48, *p* < .001, ${\eta }_{p}^{2}=.02$, but no significant interaction between gender and age group, *F*(1, 1,334) = .08, *p* =.78, ${\eta }_{p}^{2}=.00$. Means and *SD*s for the groups by gender and age-groups are given in Table 6. On average, younger males scored lower on the total scales and subscales when adjusting for group affiliation.

**Table 6:** Means, SDs of CRIES-13, CRIES-8 and three sub-scales of the measure by group, gender and age-groups.

		Community				At-risk				Total			
		Males		Females		Males		Females		Males		Females	
		*N*	M (*SD)*	*N*	M (*SD)*	*N*	M (*SD)*	*N*	M (*SD)*	*N*	M (*SD)*	*N*	M (*SD)*
**CRIES-13**	Younger	114	19.14 (11.09)	175	19.81 (10.01)	156	23.53 (10.71)	311	27.74 (11.23)	270	21.79 (11.04)	486	24.88 (11.45)
	Older	114	23.73 (10.44)	159	23.30 (11.29)	83	25.19 (10.32)	230	30.04 (13.64)	197	24.35 (10.39)	389	28.10 (12.93)
**CRIES-8**	Younger	114	13.67 (7.84)	175	13.74 (7.24)	156	16.03 (7.41)	311	18.58 (8.04)	270	15.03 (7.67)	486	24.88 (11.45)
	Older	114	16.46 (7.89)	159	17.25 (8.06)	83	17.83 (7.59)	230	20.09 (9.48)	197	17.04 (7.78)	389	18.93 (9.02)
**Intrusion**	Younger	114	6.72 (4.16)	175	7.32 (4.43)	156	8.14 (4.82)	311	9.05 (4.84)	270	7.54 (4.59)	486	8.43 (4.76)
	Older	114	8.30 (4.66)	159	8.07 (4.59)	83	9.61 (4.35)	230	10.31 (5.34)	197	8.85 (4.57)	389	9.39 (5.16)
**Avoidance**	Younger	114	6.95 (5.05)	175	6.42 (4.76)	156	7.89 (5.15)	311	9.52 (5.29)	270	7.49 (5.12)	486	8.41 (5.31)
	Older	114	8.17 (5.58)	159	9.18 (5.17)	83	8.21 (5.16)	230	9.77 (5.68)	197	8.19 (5.39)	389	9.53 (5.69)
**Arousal**	Younger	114	5.75 (4.80)	175	6.07 (4.55)	156	7.50 (5.11)	311	9.16 (5.24)	270	6.76 (5.05)	486	8.05 (5.21)
	Older	114	7.26 (4.43)	159	8.05 (4.97)	83	7.36 (4.54)	230	9.95 (5.94)	197	7.35 (4.47)	389	9.17 (5.63)

**Notes.**

CRIES-13 13-item Children Revised Impact of Event Scale CRIES-8 8-item Children Revised Impact of Event Scale

Differences on the three sub-scales were tested separately. For Intrusion, there was no significant main effect of gender, *F*(4, 1,337) = 3.42, *p* = .065, ${\eta }_{p}^{2}=.01$, but the effect for age-group was significant, *F*(4, 1,337) = 22.84, *p* < .001, ${\eta }_{p}^{2}=.02$. The interaction between gender and age group was not significant, *F*(4, 1,337) = .94, *p* = .33, ${\eta }_{p}^{2}=.01$. For the Avoidance sub-scale there were significant main effects for both gender, *F*(4, 1,337) = 9.48, *p* < .01, ${\eta }_{p}^{2}=.01$, and age-group, *F*(4, 1,337) = 11.55, *p* < .001, ${\eta }_{p}^{2}=.01$. However, the interaction between gender and age group was not significant, *F*(4, 1,337) = .19, *p* = .66, ${\eta }_{p}^{2}=.01$. Similarly, for the Arousal sub-scale, main effects for both gender, *F*(4, 1,337) = 12.31, *p* < .001, ${\eta }_{p}^{2}=.01$ and age-group, *F*(4, 1,337) = 49.70, *p* < .001, ${\eta }_{p}^{2}=.04$ were significant, but interaction between gender and age group was not significant, *F*(4, 1,337) = .38, *p* = 54.01, ${\eta }_{p}^{2}=.01$.

## Discussion

The current study reported on the psychometric properties of a Bangla language translation of the CRIES (both 13-item and 8-item versions) among a large sample of children and adolescents from community and social support centres in Bangladesh. Overall, the properties of both versions were found to be solid and broadly consistent with data from other translations of this measure.

The factor structure of the Bangla CRIES was consistent with previous findings that have demonstrated both a simple, three inter-correlated factor structure (e.g., with flood affected Chinese children, Chen et al., 2012) and a higher order three-factor structure solution (e.g., with earthquake affected Greek children, Giannopoulou et al., 2006b). Given that a simple three-factor structure is the more parsimonious solution, our data are more consistent with the former results, albeit that allowing the error terms of two items to correlate improved the fit even more. Overall, model fit indices were within acceptable ranges, however at the individual item level some items showed relatively low relationships with their respective factor (Items 3, 11, and 12). Nonetheless, we do not recommend removal of these items since the *R*^2^ values are all above .02 (Hooper, Coughlan & Mullen, 2008) and conceptually they provide a broader coverage of the relevant construct. In general, the arousal factor (.36–.47) did appear to be the weakest of the three subscales, which is consistent with previous research (Giannopoulou et al., 2006b). Therefore, future work may benefit from identification of stronger items reflecting the arousal symptoms of PTSD. However, the overall factor structure suggests that items on the CRIES sufficiently represent symptoms related to post-trauma reactions among children from Bangladesh, further supporting the universality of these symptoms (Goenjian et al., 1995; Smith et al., 2003).

The factor structure of the measure was largely consistent across various subgroups of children, including younger and older as well as females and males both within community and at-risk samples, as the model fit indices were within expected ranges. However, tests of model invariance indicated some significant differences between factor structures for particular subgroups suggesting some minor differences in the ways in which younger/older and male/female children verbalize or express PTSD symptoms. The differences between groups may be due to common response patterns, for example young females with limited literacy might respond more consistently with each other than with the broader population (Gregorich, 2006). These differences may also be reflected in the differences between subgroups on mean scores. On the other hand, the factor structure for the CRIES appeared largely similar for both community and at-risk children, supporting the universal characteristics of post-trauma symptoms irrespective of the types of traumatic exposure. The breadth of the sample in this study adds to the existing literature, which has mostly been conducted on samples following a specific type of traumatic experience, for instance, war (Smith et al., 2003), earthquake (Giannopoulou et al., 2006b), or flood (Zhang et al., 2011).

The data demonstrated that both versions of the CRIES showed good reliability when used with Bangla-speaking children and adolescents. Internal consistencies for the full 13-item and 8-item CRIES and also each sub-scale were acceptable and similar to findings from other cultures (e.g., Dyregrov, Kuterovac & Barath, 1996; Smith et al., 2003; van der Kooij et al., 2013). Test-retest reliability in our study showed acceptable stability of the measures although the modest results were not as strong as stability reported in some previous research (van der Kooij et al., 2013). Obtaining low levels of alpha is common for scales with very few items. Studies using the CRIES across various countries have found similar alpha values for the subscales to those found in the current study. Clearly, results from the sub-scales should be interpreted with caution and should not be used independently for diagnostic purposes.

As expected, the measure correlated highly with measures of anxiety and depression (Table 6) which is consistent with the results found by Lau et al. (2013) with Chinese adolescents affected by earthquake. Among the three sub-scales, arousal showed higher correlations with the other measures which is also consistent with findings by Lau et al. The moderate correlations with all total and sub-scales of the CRIES with the SCAS-20 and SMFQ indicate that although PTSD is related to both anxiety and depression, it can be identified as a construct that is distinct from both (Yule & Williams, 1990). Importantly, the CRIES-13 and CRIES-8 were able to discriminate between children from the general community and those residing in social support centres. Given that the children from support centres are considerably more likely to have experienced a large number of traumatic events (F Deeba & RM Rapee, 2014, unpublished data), these children were also at likely higher risk for PTSD and related difficulties. Therefore, these results indicate that the Bangla version of the CRIES is able to identify children who are at increased risk for PTSD, demonstrating its construct validity. Unfortunately, it was not possible in this study to obtain actual clinical diagnoses on any groups of children and therefore these conclusions about validity are based on at-risk status rather than clinical status necessitating caution in their interpretation. The lack of a clinically diagnosed group with PTSD also means that we were not able to evaluate diagnostic cut-off scores for the CRIES (Children and War Foundation, 2005) among this Bangladeshi group of young people. Examination within other samples (e.g., Australian children; Dow et al., 2012) has suggested different cut-off scores to those originally suggested by Perrin, Meiser-Stedman & Smith (2005) based on data from children in the UK. Therefore, further research is necessary to determine the best cut-off scores to identify clinical cases among children from Bangla speaking communities.

Among the Bangladeshi sample, females and older children obtained higher scores on both versions of the CRIES than males, results that are consistent with other studies (Stallard, Velleman & Baldwin, 1999; Voges & Romney, 2003). From factor analysis it seems that our participants’ primary responses to trauma are reflective of the three-factor structure of PTSD symptom clusters as represented in DSM-IV (American Psychiatric Association, 1994). However, one of our findings is most interesting in the sense that there were gender differences on both avoidance and arousal sub-scales but not on intrusion. It is possible that these results show the universality of intrusion as a characteristic of PTSD (Green et al., 1991) given that girls scored higher on the other two symptom clusters but not on intrusion. As the higher scores from females on avoidance and arousal are more consistent with typical findings that females tend to report higher levels of psychological reactions to -traumatic events (Giaconia et al., 1995), as well as more generally higher levels of anxiety and depression (Davis & Siegel, 2000). Moreover, in a patriarchal culture like Bangladesh it is also likely males will report less avoidance and arousal symptoms due to the influence of social roles.

These gender and age differences are consistent with broader findings relating to gender and age differences in the experience of traumatic events and reporting of stress reactions. Many studies have shown that although males experience a greater number of traumatic events, females and older children report higher levels of classic symptoms of PTSD as reactions to these events (Dyregrov, Kuterovac & Barath, 1996; Giannopoulou et al., 2006b; Yule, 1999). Other authors have suggested that the three main criteria of PTSD better represent older children’s post-traumatic stress reactions than younger (Broman-Fulks et al., 2009). This indicates the need for extensive studies on stress reactions in younger children in future studies. However, before administering the scale with any children, researchers should take care to familiarize themselves with the symptoms of PTSD in children and adolescents as per diagnostic criteria. Given the large and diverse sample of Bangladeshi children included in this study, the scores obtained by various sub-groups (such as different ages, genders or risk status) will allow mental health professionals or researchers in Bangladesh to compare their samples with the relevant subgroup.

One of the main limitations of this study was the lack of diagnostic data. Diagnoses provide the gold standard against which to evaluate the validity of a measure of psychopathology (Jaeschke, Guyatt & Sackett, 1994) and the lack of this standard means that it was not possible to determine the ability of the CRIES to identify likely cases. Further, determining the psychometric properties of the 8-item version of the CRIES based on completion of the 13-item version may not provide exactly the same psychometric properties that might be found with use of the 8-item version alone. Therefore, although the properties looked promising, they need to be replicated in future studies that use only the 8-item version of the Bangla CRIES. This limits the conclusions we can draw regarding the use of the Bangla CRIES for population screening (Dow et al., 2012; Kenardy, Spence & Macleod, 2006).

Nevertheless, the current data suggest that the Bangla CRIES is a potentially useful instrument to assess post-trauma reactions among young Bangladeshi people. Given the impact on functioning of experiences with severe trauma among children (Abdel-Mawgoud & Al-Haddad, 1997; Almqvist & Brandell-Forsberg, 1997; Caffo, Forresi & Lievers, 2005; Laor et al., 1996; Terr, 1983), identification of distress in response to these experiences as early as possible is important in a developing country like Bangladesh. These measures should be of value in both clinical settings and at a community level to assess the need for services. The short CRIES-8 is likely to be especially useful in acute crisis situations. The particular strengths of the CRIES, including brevity, simplicity, and low cost, means that this measure will be of tremendous value for identification, assessment, and appropriate intervention for young people in Bangladesh. Such a tool will be useful for professional mental health workers as well as semi-skilled professionals who work with emergencies or in crisis-affected areas.

## Supplemental Information

10.7717/peerj.536/supp-1 Supplemental Information 1 ”Invitation to participate in a study” letter

10.7717/peerj.536/supp-2 Supplemental Information 2 dataset_onCRIES_Bangladeshi_children
