# Supplementary material for: Psychometric properties of the Children’s Revised Impact of Events Scale (CRIES) with Bangladeshi children and adolescents
Source: PeerJ. 2014 Aug 26;2:e536. doi: 10.7717/peerj.536 (PMC4157240; doi:10.7717/peerj.536)
Supplement: Supplemental Information 1 [file peerj-02-536-s001.pdf]

MACQUARIE UNIVERSITY
CENTRE FOR EMOTIONAL HEALTH

Department of Psychology,

Sydney. NSW. 2109. Australia

Invitation to participate in a study of child and adolescent mental health

Date…………..

Dear Parent/Guardian,

We would like to invite you and your child to participate in a study on "children's thinking style after experiencing disastrous incidents". The study is being conducted by, Farah Deeba, Assistant Professor, Department of Clinical Psychology, University of Dhaka, Dhaka, phone- 9661920-49 Exten-7804, e-mail- farah.deeba@mq.edu.au. The research is being conducted to meet the requirements for the degree of PhD in Psychology under the direct supervision of Prof. Ronald M. Rapee, Director, Emotional Health Centre, Department of Psychology, Building- C3A 721, Macquarie University, NSW, Australia.

The study will need you and your child to visit the place assigned by the organization that you first contacted. Your child will participate in a session with other children where they will be requested to complete 4 questionnaires that will take approximately 40-50 minutes. Your child's participation in this study is entirely voluntary and you have no bound to give consent if you do not want. An information sheet which contains information about impact of traumatic events, addresses of available mental health services, the details of the present study and a parent/guardian consent form are provided here. After you have read/heard about the study, and if you agree, then I will ask your daughter/son for their agreement as well. Both of you have to agree independently before we begin. You do not have to decide today whether or not you agree to have your child participate in this research. If you decide to participate at a later time please contact Farah Deeba by phone or e-mail.

The questionnaires that you and your child will fill out are confidential. All information related to the child and family will be kept confidential, unless it is unethical to do so. No one will be identified in any reporting, presentations or in publications. Only Prof. Rapee and Farah Deeba will have access to the questionnaires. The research team will keep the responses on the questionnaires for 5-7 years for analysis and reporting purposes and they will be destroyed after that.

Thinking about a stressful life event in the past may create feelings of distress for the moment in your child. Your child will be explained before starting the assessment that if s/he feels distressed during the session, to report to Farah Deeba. S/he may withdraw from completing the questionnaires. If you find that your child is feeling distressed after you have left the session, **please contact Farah Deeba (01715-696833)**. Alternatively you may contact the clinical psychology department or any of the contacts given in the information leaflet.

Your child will receive a gift of two prize bonds (value of 200BDT) to thank them for participation in the study. You will also be reimbursed for your travel and time in completing the questionnaires related to the child(ren). There will be no immediate and direct benefit to your child or to you, but your child's participation is likely to help us find out more about the mental health aspects of young individuals. You will be given information on the psychological impacts of the exposure to the traumatic events and also with the addresses of available support systems in our country. With the information you will be able to evaluate your child's mental health conditions as a result of the trauma and will be able to get in touch with an appropriate service provider.

This information sheet is for you to keep.[1]

[1] *The ethical aspects of this study have been approved by the Macquarie University Human Research Ethics Committee. If you have any complaints or reservations about any ethical aspect of your participation in this research, you may contact the Committee through the Director, Research Ethics (telephone [02] 9850 7854, fax [02] 9850 8799, email: ethics@mq.edu.au. Any complaint you make will be treated in confidence and investigated, and you will be informed of the outcome.*

MACQUARIE UNIVERSITY
CENTRE FOR EMOTIONAL HEALTH

Department of Psychology,

Sydney. NSW. 2109. Australia

PART II: Certificate of Consent

Informed Consent Form for parents/guardian of children and adolescent girls and boys participating in
The research titled, "*Trauma-Specific negative cognitions in children and adolescents*".

I, ... voluntarily consent for my child ……………………………………who is aged, ………… years, to participate in the research study entitled: ***Trauma-specific negative cognitions in children and adolescents.***

In giving my consent I acknowledge that: I have read all the information provided in the previous sections, or it has been read out to me. I have understood the procedures and protocols involved in the study and I have been given the opportunity to ask questions about it. I have been asked to give consent for my daughter/son to participate in this research study which will involve her completing a few questionnaires. I understand that information given by my child is confidential and no personal information regarding identity of my child has been kept. I understand that s/he will also be asked to give permission and that her/his wishes will be respected. I have been informed of any risks. I am aware that there may be no benefit to either my child or me personally. I understand that I have the right to withdraw her/him from the study at any time without any negative consequences.

Name of Parent or Guardian ___________________
Signature of Parent or Guardian___________________
Date ____________________________
Day/month/year

Name of Child or adolescent ___________________
Signature of Child or adolescent___________________
Date ____________________________
Day/month/year

If illiterate
I have witnessed that the consent form has been read out to the parent of the child. The individual was given opportunity to ask questions as well. I confirm that the individual has given consent on his/her free will.
Print name of witness______________________ AND Thumb print of participant
Signature of witness _______________________
Date _________________________
Day/month/year

Print name of researcher_________________________
Signature of researcher __________________________
Date ____________________________
Day/month/year

MACQUARIE UNIVERSITY
CENTRE FOR EMOTIONAL HEALTH

Department of Psychology,

Sydney. NSW. 2109. Australia

PART II: Certificate of Consent[2]

Informed Consent Form for parents/guardian of children and adolescent girls and boys participating in
The research titled, "*Trauma-Specific negative cognitions in children and adolescents*".

I, ... voluntarily consent for my child ……………………………………who is aged, ………… years, to participate in the research study entitled: ***Trauma-specific negative cognitions in children and adolescents.***

In giving my consent I acknowledge that: I have read all the information provided in the previous sections, or it has been read out to me. I have understood the procedures and protocols involved in the study and I have been given the opportunity to ask questions about it. I have been asked to give consent for my daughter/son to participate in this research study which will involve her completing a few questionnaires. I understand that information given by my child is confidential and no personal information regarding identity of my child has been kept. I understand that s/he will also be asked to give permission and that her/his wishes will be respected. I have been informed of any risks. I am aware that there may be no benefit to either my child or me personally. I understand that I have the right to withdraw her/him from the study at any time without any negative consequences.

Name of Parent or Guardian __________________
Signature of Parent or Guardian___________________
Date ___________________________
Day/month/year

Name of Child or adolescent __________________
Signature of Child or adolescent___________________
Date ___________________________
Day/month/year

If illiterate
I have witnessed that the consent form has been read out to the parent of the child. The individual was given opportunity to ask questions as well. I confirm that the individual has given consent on his/her free will.
Print name of witness_____________________ AND Thumb print of participant
Signature of witness ______________________
Date ________________________
Day/month/year

Print name of researcher________________________
Signature of researcher _________________________
Date ___________________________
Day/month/year
(Researcher's copy)

[2] *A copy of this Informed Consent Form has been provided to the parent/guardian of the child*
